# Effect of autologous hematopoietic stem cell transplant on the development of second primary malignancies in multiple myeloma patients

**DOI:** 10.1038/s41408-020-00400-4

**Published:** 2021-01-07

**Authors:** A. S. Rosenberg, A. Brunson, J. Tuscano, B. A. Jonas, R. Hoeg, T. Wun, T. H. M. Keegan

**Affiliations:** 1 Davis School of Medicine, Center for Hematology and Oncology Outcomes Research and Training (COHORT), University of California, Sacramento, CA USA; 2 Davis Comprehensive Cancer Center, University of California, Sacramento, CA USA; 3 Northern California Department of Veterans Affairs, Sacramento, CA USA

**Keywords:** Myeloma, Risk factors, Cancer prevention

## Abstract

Autologous stem cell transplant (aHSCT) is associated with improved survival for multiple myeloma (MM) patients but may be associated with second primary malignancy (SPM) development. Using the California Cancer Registry linked to statewide hospitalization data, we determined the cumulative incidence (CMI) of SPMs more than 1 year after MM diagnosis, accounting for the competing risk of death. AHSCT recipients were matched 1:2 to non-aHSCT patients. Adjusted hazard ratios (aHR) were estimated using the Fine and Gray method. Among 16,331 patients, 933 (5.7%) developed a SPM more than 1 year after diagnosis. The 10-year CMI of developing any SPM was 6.6%, 5.7% for solid tumor SPM and 0.9% for hematologic malignancies. The 10-year CMI of developing any SPM was similar among aHSCT [9.1% (7.7–10.7%)] and non-aHSCT [7.5% (6.5–8.6%)] (*P* = 0.26) recipients and there was no difference in solid-tumor SPMs (*P* = 0.98). The 10-year CMI of hematologic SPMs was higher among aHSCT recipients [2.1% (1.4–2.9%) vs. 0.8% (0.5–1.2%); *P* = 0.005], corresponding to a 1.3% absolute increase and an aHR of 1.51 (1.01–2.27). Ten-year myeloma-specific and non-cancer mortality rates were 59% (58.2–60.0%) and 18.1% (17.4–18.8%), respectively. Although aHSCT was associated with a small increase in hematologic SPMs, mortality was driven by MM and non-cancer causes.

## Introduction

Multiple myeloma survival has improved dramatically over the last 20 years due to adoption of autologous stem cell transplant (aHSCT) and multiple new drug classes for treatment^1–7^. In the era of novel agents, randomized phase 3 trials have confirmed survival benefits to aHSCT^8–12^. As the survival of multiple myeloma patients improves, late effects are now being recognized and need to be addressed^13,14^. Multiple myeloma patients have been found to be at an increased risk of second primary malignancies (SPMs) compared to the general population^15–27^, with treatments, notably the immunomodulatory agents and alkylator based chemotherapy, likely contributing to this increased risk^16,20,22,23,28–30^.

Randomized controlled trials (RCTs) of aHSCT use in the modern treatment era have compared early to delayed aHSCT, but have not compared SPM development in these two arms^8–10^. Lenalidomide maintenance trials post-aHSCT have found higher SPM rates in the maintenance arms, but cannot address the potential additional risk contributed by high dose chemotherapy^19,31–33^.

Cancer registry-based studies have used diagnosis date as a proxy for exposure to aHSCT with conflicting results^23,34^. Analyses of large stem cell transplant registries comparing SPM incidence among aHSCT recipients to general population cancer incidence rates found the risks of acute lymphoblastic leukemia, acute myeloid leukemia, Hodgkin lymphoma were increased^15,20^. However these studies relied on comparisons of rates in different registries, and cross-registry comparisons may be confounded. No prior population level studies have directly compared the incidence of SPMs in myeloma patients, who did and did not undergo an aHSCT.

To address these gaps in the literature, we utilized longitudinal data on multiple myeloma patients from the population-based California Cancer Registry (CCR) linked to California Patient Discharge Database (PDD) and Ambulatory Surgery (AS) database to identify those undergoing aHSCT. Findings from this study will provide valuable insight into the potential late effects of aHSCT to better inform patient decisions.

## Methods

### Databases

This retrospective observational cohort study utilized linked data between the CCR and California PDD and AS databases, which are maintained and previously linked by The Office of Statewide Health Planning and Development. The CCR is a statewide population-based cancer surveillance system collecting cancer incidence and mortality information since 1988; it captures >98% of all cancer diagnoses in the state excluding non-invasive squamous and basal cell carcinomas of the skin. From the CCR, we obtained date of diagnosis, initial course of treatment, and patient demographics, including race/ethnicity, gender, age, location of residence (rural vs. urban), marital status, neighborhood socioeconomic status^35^, and insurance type at time of diagnosis^36^. The PDD captures information on all discharges from non-federal hospitals in California since 1991. Beginning in 2005, the AS database, including all hospital associated AS facilities, has also been mandated. The databases were linked at the patient level using the record linkage number (RLN), an encrypted form of social security number. The RLN allows serial linking of multiple hospitalization records over time. Patients who did not have a RLN (11%) or were only reported by Department of Veterans Affairs (which does not send data to the PDD or AS, and provides data to CCR but cannot be disclosed for research purposes) were excluded. Both PDD and AS include up to 25 diagnoses and up to 21 procedures associated with each hospitalization, coded using the International Classification of Disease, Ninth Revision, Clinical Modification (ICD-9-CM) in the PDD and Current Procedural Terminology (CPT) in the AS. Each procedure code has a date, and were used to ascertain aHSCT and comorbidities.

### Patients

Between 1991–2014, patients with first primary multiple myeloma patients were identified in CCR using ICD-O-3 histology code 9732^37^. To account for surveillance bias and immortal time bias, all patients had to have at least 1 year of follow-up time recorded, and all patients who developed a SPM during the first year after diagnosis were excluded.

### Exposure

Only first aHSCT use was examined, and was considered present if it was identified in either the CCR, PDD or AS, and was considered “early”, if performed within 12 months of diagnosis, or “late”, if greater than 12 months, as done previously^12^. Year of diagnosis was categorized into four treatment eras (1991–1997: pre-immunomodulatory (imid) and infrequent aHSCT use, 1998–2002: increased imid and aHSCT use, 2003–2007: introduction of proteasome inhibitors (PI) and second generation imids, 2008–2013: modern combination imid and PI more commonly used)^34^. Comorbidities were captured in the PDD up to 2 years prior to the multiple myeloma diagnosis date. They were identified using the Elixhauser index, excluding cancer^38^, and categorized as no admissions in PDD within the 2 prior years (and thus no information), 0 comorbidities, 1–2 comorbidities, and ≥3 comorbidities. Neighborhood socioeconomic status (nSES) is measured at the neighborhood level by the CCR, and divided into quintiles^35^. First-course chemotherapy and radiation are captured as a binary variables without details of regimen or dosing.

### Outcome

First SPM was obtained from the CCR and classified using SEER site recode. Non-invasive squamous and basal cell carcinomas of this skin are not reportable to the CCR and were not included, while in situ breast cancers were included. No additional malignancies beyond the second were considered in this study. First SPM was evaluated as overall, solid, and hematologic. Cause specific mortality was assessed using cause of death ascertained from death certificates by CCR.

### Statistics

The cumulative incidence (CMI) of developing any SPM, solid tumors, and hematologic malignancies, and associated 95% confidence intervals were computed, accounting for the competing risk of death. The cumulative incidence of multiple myeloma specific mortality, non-cancer mortality, and SPM development were compared using the competing risk framework. To compare the conditional cumulative incidence of SPMs between aHSCT recipients and those without aHSCT, patients were matched 1:2 on sex, age +/− 3 years, year of diagnosis +/− 2 years, race/ethnicity, nSES status (quintiles), Elixhauser comorbidity index (NA, 0, 1–2, 3+), and follow-up time (Supplemental Table S1). Multivariable Cox proportional hazards regression models, using the methods of Fine and Gray to account for competing risk of death, estimated the effect of aHSCT on the risk of SPM development accounting for baseline demographics. Autologous HSCT was included as a time dependent covariate. The proportional hazard assumption was assessed using Schoenfeld residuals^39^, and models were stratified for variables that did not meet the proportional hazards assumption. All calculations were using SAS (version 9.4, Cary, NC).

This study was reviewed by the Committee of the Protection of Human Subjects, which serves as the Institutional Review Board for the California Health and Human Services Agency, and by the University of California Davis Institutional Review Board.

## Results

### Patient characteristics

Between 1991 and 2013, 16,331 patients with multiple myeloma were identified within the CCR and met the inclusion criteria (Table 1). The median age at diagnosis was 66 years. There was a slight male predominance (54%). Non-Hispanic whites made up the largest proportion of patients (59%), followed by Hispanics (18%), African Americans (13%), and Asians (9%). Autologous HSCT was utilized in 19.6% of newly diagnosed patients, more commonly among men, younger patients, those with fewer comorbidities, higher neighborhood socioeconomic status and private insurance, and less commonly among African Americans and those with Medicare, no or unknown insurance. The use of aHSCT increased over time.

**Table 1:** Baseline multiple myeloma characteristics by autologous stem cell transplant (aHSCT) among 1-year survivors of first primary multiple myeloma in California, 1991–2013.

	All	aHSCT			No aHSCT		
	*N*	%	*N*	col %	*N*	col %	*P*-value
All	16,331	100.0	3202	100.0	13,129	100.0	
*Gender*							
Male	8767	53.7	1868	58.3	6899	52.5	<0.0001
Female	7564	46.3	1334	41.7	6230	47.5	<0.0001
*Race/Ethnicity*							
Non-Hispanic White	9591	58.7	1901	59.4	7690	58.6	0.4117
African–American	2115	13.0	358	11.2	1757	13.4	0.0009
Hispanic	2954	18.1	649	20.3	2305	17.6	0.0004
Asian/Pacific Islander	1467	9.0	283	8.8	1184	9.0	0.7495
Other/Unknown	204	1.2	11	0.3	193	1.5	<0.0001
*Age diagnosis*							
Age <40	325	2.0	137	4.3	188	1.4	<0.0001
40–49	1504	9.2	672	21.0	832	6.3	<0.0001
50–59	3569	21.9	1322	41.3	2247	17.1	<0.0001
60–69	4744	29.0	982	30.7	3762	28.7	0.0244
70–79	4257	26.1	88	2.7	4169	31.8	<0.0001
80–89	1815	11.1	1	0.0	1814	13.8	<0.0001
≥90	117	0.7			117	0.9	<0.0001
*Treatment era of diagnosis*							
1991–1997	3949	24.2	372	11.6	3577	27.2	<0.0001
1998–2002	3268	20.0	585	18.3	2683	20.4	0.006
2003–2007	3856	23.6	971	30.3	2885	22.0	<0.0001
2008–2013	5258	32.2	1274	39.8	3984	30.3	<0.0001
1st Course of treatment							
Chemotherapy							
Yes	12,071	73.9	3007	93.9	9064	69.0	<0.0001
No	4023	24.6	187	5.8	3836	29.2	<0.0001
Unknown	237	1.5	8	0.2	229	1.7	<0.0001
Radiation							
Yes	3,824	23.4	946	29.5	2878	21.9	<.0001
No	12,499	76.5	2256	70.5	10,243	78.0	<.0001
Unknown	8	0.0			8	0.1	0.1624
Neighborhood socioeconomic status (quintiles)							
1-Lowest	2374	14.5	366	11.4	2008	15.3	<0.0001
2	2854	17.5	476	14.9	2378	18.1	<0.0001
3	3333	20.4	655	20.5	2678	20.4	0.9414
4	3755	23.0	804	25.1	2951	22.5	0.0015
5-Highest	4015	24.6	901	28.1	3114	23.7	<0.0001
Insurance coverage							
No insurance/Self pay	190	1.2	22	0.7	168	1.3	0.0051
Private insurance	7256	44.4	2154	67.3	5102	38.9	<0.0001
Medicaid/Gov	1255	7.7	314	9.8	941	7.2	<0.0001
Medicare	5570	34.1	586	18.3	4984	38.0	<0.0001
Unknown insurance	2059	12.6	126	3.9	1933	14.7	<0.0001
Comorbidities^a^							
No admissions	7445	45.6	1613	50.4	5832	44.4	<0.0001
0 Comorbidities	1511	9.3	412	12.9	1099	8.4	<0.0001
1–2 Comorbidities	3614	22.1	700	21.9	2914	22.2	0.6833
≥3 Comorbidities	3761	23.0	477	14.9	3284	25.0	<0.0001
SPM site							
All	933	5.7	186	5.8	747	5.7	0.7945
Digestive system	184	1.1	22	0.7	162	1.2	0.0025
Breast	97	0.6	18	0.6	79	0.6	0.7195
Respiratory system	119	0.7	19	0.6	100	0.8	0.2459
Leukemia	81	0.5	32	1.0	49	0.4	<0.0001
Lymphoma	40	0.2	6	0.2	34	0.3	0.4245
MDS	31	0.2	8	0.2	23	0.2	0.4054
Female genitals	36	0.2	9	0.3	27	0.2	0.4379
Urinary system	66	0.4	7	0.2	59	0.4	0.0491
Male genitals	115	0.7	31	1.0	84	0.6	0.0442
Brain and other CNS	7	0.0	–	–	7	0.1	0.1851
Endocrine system	19	0.1	6	0.2	13	0.1	0.1993
Soft tissue	4	0.0	2	0.1	2	0.0	0.1315
Bone and joint	1	0.0	–	–	1	0.0	0.6176
Oral cavity system	29	0.2	9	0.3	20	0.2	0.1286
Skin	75	0.5	15	0.5	60	0.5	0.9884
Mesothelioma	4	0.0	1	0.0	3	0.0	0.7994
Miscellaneous	25	0.2	1	0.0	24	0.2	0.0432

*nSES* neighborhood socioeconomic status, *SPM* second primary malignancy, *MDS* myelodysplastic syndrome.

^a^Comorbidities were calculated within 2 years prior to multiple myeloma diagnosis.

### Second primary malignancy (SPM)

SPMs were identified in 933 (5.7%) patients (Table 1). The median time from the diagnosis of MM to the diagnosis of SPMs was 3.8 years (range 1.0–24.5 years). Solid tumors were more commonly diagnosed than hematologic malignancies, with gastrointestinal, breast, and male genitourinary cancers being the most common. Among hematologic malignancies, leukemia was the most common, accounting for 8.7% of SPMs. Among these, acute myeloid and acute lymphoid leukemia were the most commonly diagnosed accounting for 58% and 21% of leukemia diagnoses, respectively. Myelodysplastic syndrome was captured by the CCR starting in 2002, accounting for 3.3% of all SPMs.

**Fig. 1:**
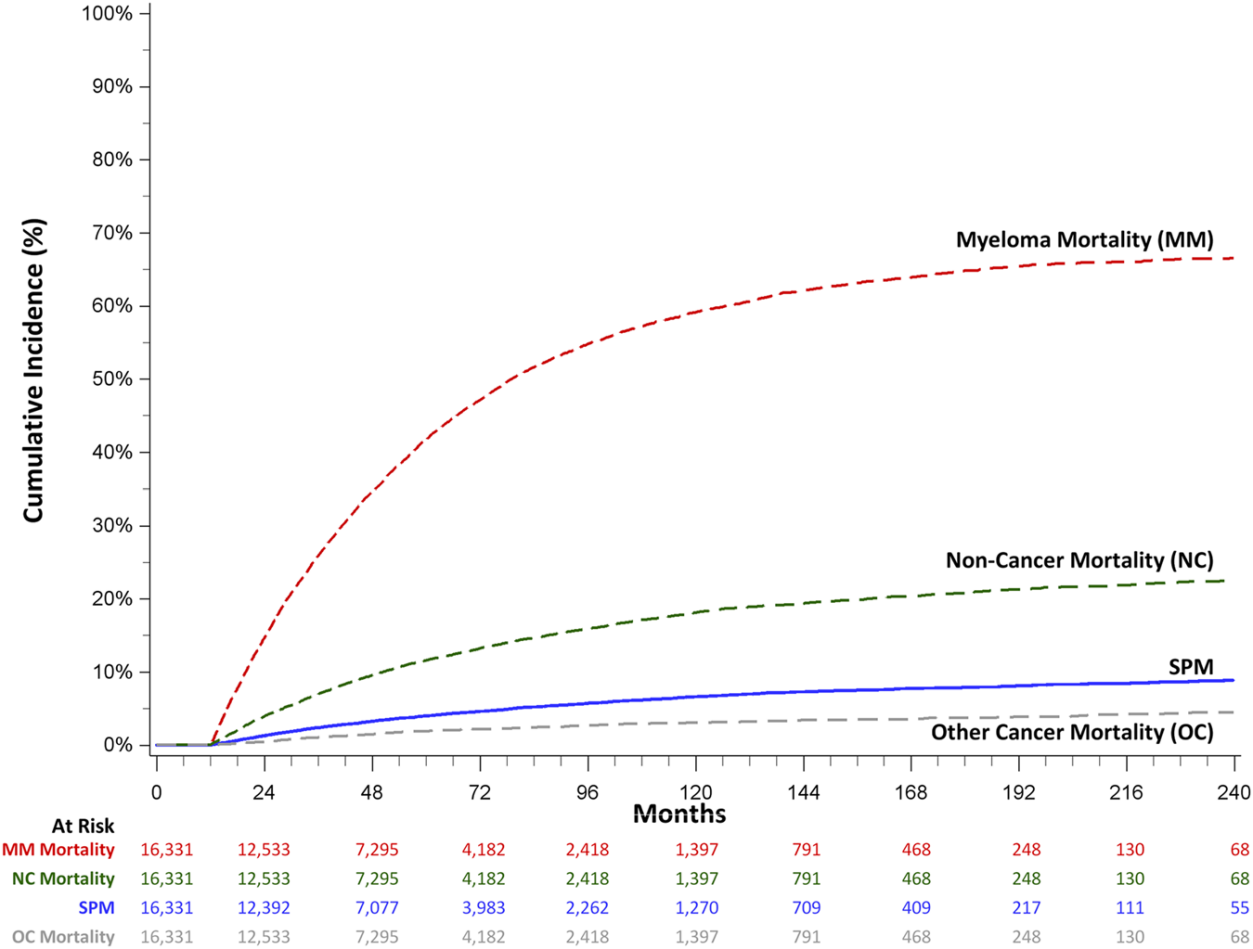
Cumulative incidence of mortality and second primary malignancy (SPM) among 1-year survivors of first primary multiple myeloma in California, 1991–2013. The cumulative incidence of second primary malignancy (SPM) development accounting for the competing risk of death from multiple myeloma (MM), non-cancer related mortality (NC) and other cancer mortality (OC).

The cumulative incidence of developing any SPM was 4.0% (3.7–4.3%) at 5 years and 6.6% (6.2–7.1%) at 10 years after diagnosis (Fig. 1). Multiple myeloma-related mortality was 41.8% (41.0–42.6%) and 59.1% (58.2–60.0%) and non-cancer related mortality was 11.6% (11.0–12.1%) and 18.1% (17.4–18.8%) at 5 and 10 years, respectively. By comparison, death attributable to SPMs were 1.9% (1.7–2.1%) and 3.0% (2.7–3.3%).

**Fig. 2:**
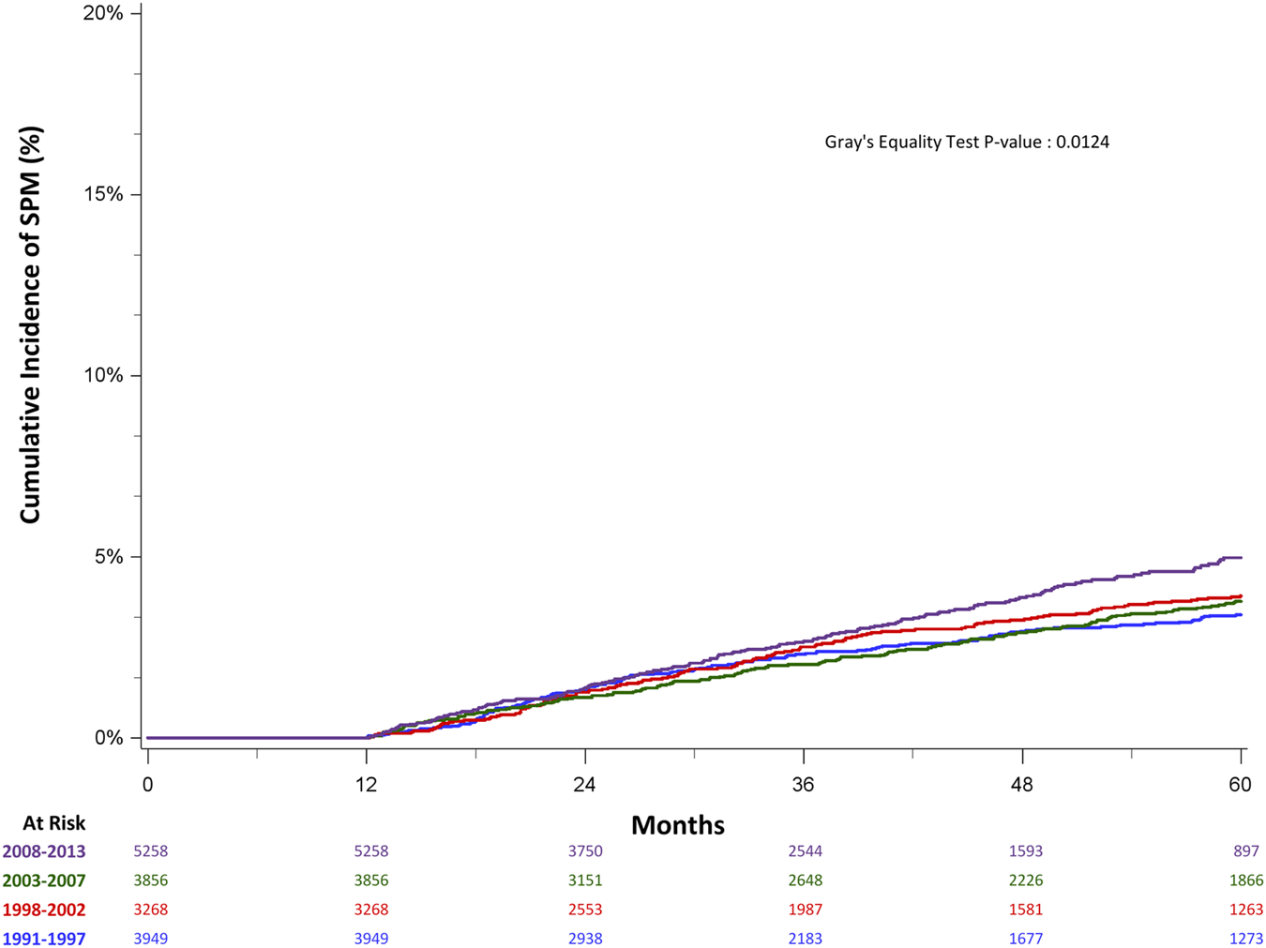
Five-year cumulative incidence of second primary malignancy by treatment era among 1-year survivors of first primary multiple myeloma in California, 1991–2013. The cumulative incidence of developing second primary malignancies in patient cohorts diagnosed between 1991–1997, 1998–2002, 2003–2007, 2008–2013, accounting for the competing risk of death. The 5 year cumulative incidence of SPM development was higher in those diagnosed between 2008–2013 [5.0% (4.3–5.8%)] compared to earlier eras: 3.8% (3.2–4.4%), 3.9% (3.3–4.6%), 3.4% (2.9–4.0%) in 2003–2007, 1998–2002, and 1991–1997.

Rates of SPM development changed during the different treatment eras. The 5-year CMI rate for patients diagnosed 2008–2013 was 5.0% (4.3–5.8%) compared to 3.8% (3.2–4.4%), 3.9% (3.3–4.6%), 3.4% (2.9–4.0%) in 2003–2007, 1998–2002, and 1991–1997, respectively (*P* < 0.001) (Fig. 2). Median time to develop a SPM was similar in the earlier three eras: 54 months, 52 months, and 53 months for those diagnosed 1991–1997, 1998–2002, and 2003–2007, respectively. In contrast, the median time to SPM in patients diagnosed 2008–2013 was 31 months, consistent with shorter follow-up time in the more recent cohort. Because myelodysplastic syndrome (MDS) was only recorded starting in 2002, we performed a sensitivity analysis excluding MDS and observed similar findings (Supplemental Fig. 1).

### Association of second primary malignancy and aHSCT use

**Fig. 3:**
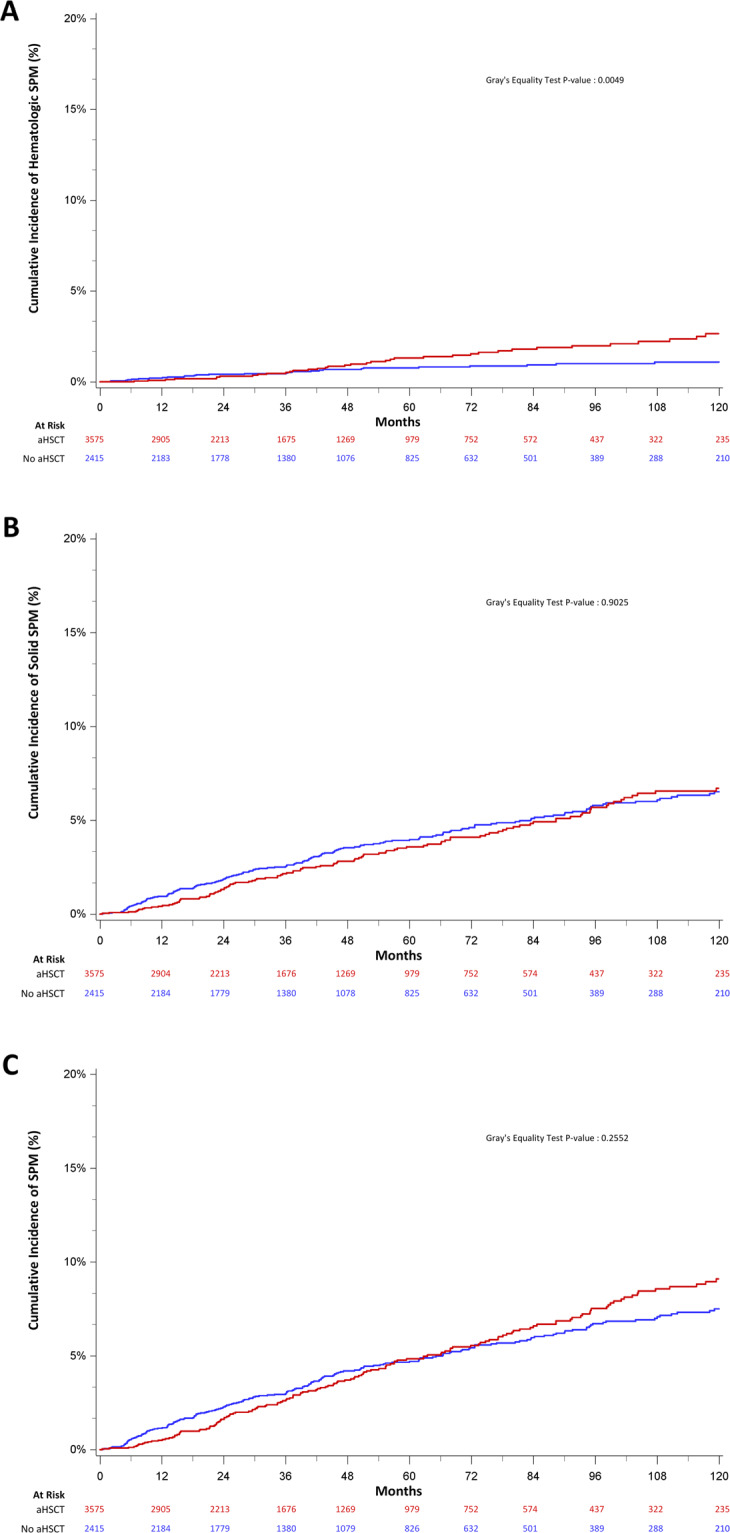
Cumulative incidence of second primary malignancy development among multiple myeloma patients with or without autologous stem cell transplant. Overall solid hematologic footnote: **A** overall second primary malignancy (SPM), **B** Solid tumor SPM, and **C** Hematologic SPM by autologous stem cell transplant (aHSCT) treatment among matched 1-year survivors with first primary multiple myeloma in California, 1991–2013. aHSCT patients were matched to two patients without aHSCT on age, year of diagnosis, race/ethnicity, nSES status comorbidities, and follow-up time. Person time was calculated from aHSCT date to SPM, death, or last known follow-up from the cancer registry.

**Fig. 4:**
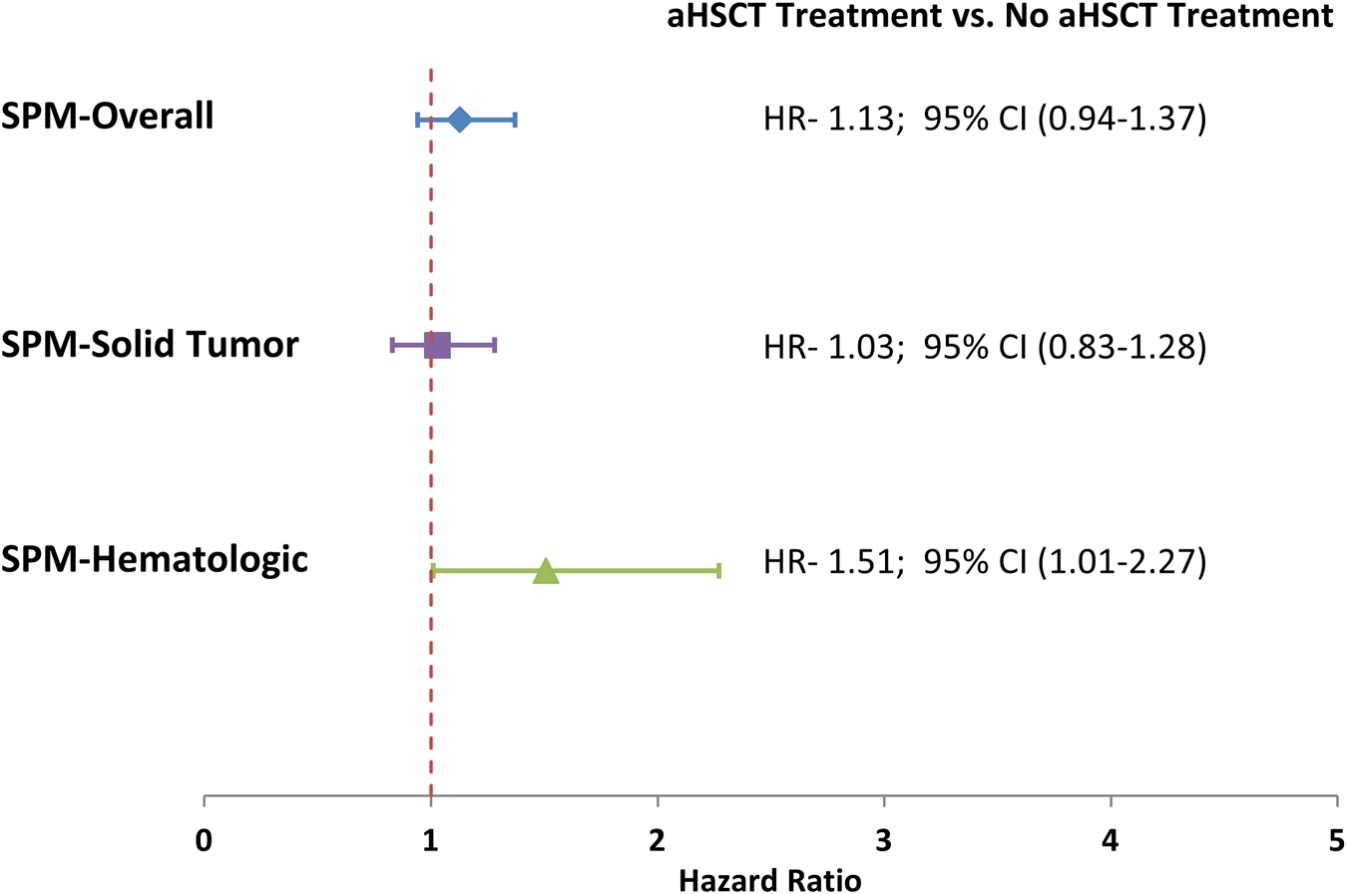
Adjusted effect of autologous stem cell transplant (aHSCT) treatment compared to no aHSCT treatment on the risk of second primary malignancy (SPM) among 1-year survivors with first primary multiple myeloma in California, 1991–2014. aHSCT was included as a time dependent covariate. Adjusted for the competing risk of death and baseline multiple myeloma characteristics (gender, race/ethnicity, age at diagnosis, treatment era of diagnosis, initial course of treatment, neighborhood socioeconomic status, comorbidities, initial health insurance, marital status, and urban vs. rural location of residence).

Patients undergoing aHSCT did not have an increased risk of any SPMs, with 5-year and 10-year CMI rates of 4.8% (3.9–5.9%) and 9.1% (7.7–10.7%) compared to 4.7% (4.0–5.5%) and 7.5% (6.5–8.6%), respectively (*P* = 0.26) among non-aHSCT patients (Fig. 3a). In multivariable analysis, this corresponded to an adjusted hazard ratio of 1.13 (0.94–1.4, *P* = 0.19) (Fig. 4). We then examined the association between aHSCT use and the development of solid tumor SPMs and hematologic SPMs. The 5-year and 10-year CMI of developing any solid tumor SPM among aHSCT recipients was 3.6% (2.8–4.5%) and 6.7% (5.5–8.1%) compared to 3.9% (3.3–4.7%) and 6.5% (5.5–7.6%) among non-aHSCT recipients (Fig. 3b) (*P* = 0.90). This corresponded to an aHR of 1.03 (0.83–1.28, *P* = 0.79) in multivariable analysis (Fig. 4). In contrast, the 5-year and 10-year CMI of developing any hematologic SPM among aHSCT recipients was 1.3% (0.9–1.9%) and 2.7% (1.9–3.7%) compared to 0.8% (0.5–1.1%) and 1.1% (0.7–1.6%) among non-aHSCT recipients (Fig. 2c; *P* = 0.005) (Fig. 3c), corresponding to an aHR of 1.51 (1.01–2.27; *P* = 0.046) (Fig. 4).

## Discussion

To our knowledge, this is the largest study examining the association between aHSCT and the development of SPMs in multiple myeloma patients. We observed that 6.6% of multiple myeloma patients developed a SPM 10 years after diagnosis and the risk of developing hematologic malignancies, but not solid tumors, was higher among those undergoing aHSCT. Specifically, we found an absolute increase of 1.3% in hematologic malignancies (from 0.8% among non-aHSCT recipients to 2.1% among aHSCT recipients). Despite this increased risk, myeloma-specific and non-cancer mortality greatly outweigh the risk of developing SPMs, and the 10 year mortality due to SPM was low, suggesting that benefits of aHSCT^8–12,40^ outweigh risks due to SPMs.

Two prior observational studies have addressed the risk of developing SPMs in multiple myeloma patients who have undergone aHSCT. In the United States, the Centers for International Blood & Marrow Research (CIBMTR) database identified 4161 aHSCT recipients between 1990–2010. After accounting for the competing risk of death, 2.6 and 5.1% developed a SPM 3 and 7 years post-aHSCT. When compared to the rates of acute myeloid leukemia and myelodysplastic syndrome development in the general population, significant increased risk was identified (standardized incidence ratios 5.19 and 85.5, respectively)^20^. While our findings were concordant, it is notable that the differential risk of developing a hematologic SPM was much lower in our study, likely due to our comparison group of multiple myeloma patients having a higher risk of developing these diseases when compared to the general population. A sub-study of the European Bone Marrow Transplant Registry identified 3204 multiple myeloma patients, of whom 135 developed SPMs. At 6 years, the cumulative incidence, accounting for the competing risk of death, was 5.3% overall (1.4% for hematologic malignancies and 3.6% for solid tumors). The current study found similar 5-year overall cumulative incidence rate of 4.0% (0.6% hematologic and 3.4% solid tumors).

Multiple prior population-based studies have examined the relationship between multiple myeloma and SPM development. An analysis of the SEER program between 1973–2008 showed that myeloma patients had a nearly 10% lower incidence of solid tumor development than the background population. In distinction, they found a more than a 60% increased incidence of developing hematologic malignancies, both myeloid and lymphoid: SIR 1.63 (1.45–1.84), similar to our findings. When SPM rates were compared across treatment eras, no change in the risk of SPM development was identified^21,23^. An analysis of the Swedish healthcare registry and a registry of MGUS patients diagnosed between 1986–2005 reported a nearly 30% increased risk of SPMs in multiple myeloma patients (SIR 1.26 [1.16–1.36]). This effect was particularly pronounced for hematologic malignancies, with a more than 2-fold increased incidence (SIR 2.04 [1.59–2.58]), and no differences in incidence was observed over time. Interestingly, MGUS patients had an increased incidence of AML, MDS, and myeloproliferative neoplasms (MPNs), implying that an underlying bone marrow process or stem cell defect may link these diagnoses, rather than treatment^26^. An analysis comparing SPM rates for MM patients in German and Swedish national cancer registries found different distributions of SPMs in the two populations—though an increased incidence of AML was observed in both^18^. Thus, factors that have yet to be elucidated are also likely involved in SPM development in multiple myeloma patients.

We found that the rates of SPM development have increased in the most recent era. However, this finding needs to be considered preliminary and hypothesis generating until more complete follow-up allows for further study. Differences in the findings of the current and prior studies may be due to differences in analytic methods, background population risk of SPM development, follow-up time, or latency periods, along with the inclusion of a more modern study population with different drug exposures, such as incorporation of post-aHSCT maintenance, and trends in cancer screening along with an increasing awareness of SPM risk in multiple myeloma patients. Future studies focusing on changing trends in SPM development are needed to identify populations at highest risk to better inform both patients and physicians, and allow for targeted screening of multiple myeloma patients.

RCTs of continuous lenalidomide therapy have observed higher SPM rates among patients undergoing lenalidomide maintenance after aHSCT, but not in transplant ineligible patients undergoing continuous lenalidomide based therapy compared to placebo^31–33,41,42^. A meta-analysis of lenalidomide trials identified patients receiving oral melphalan and lenalidomide at highest risk for developing SPMs, though those receiving intravenous melphalan and lenalidomide had numerically higher incidence of SPM development than those not receiving lenalidomide^22^. The current study found lower rates of SPM development after aHSCT than was seen in RCTs. This may be related to several factors, including higher competing risks of death in the general population compared to populations included in RCTs^43^, variable adoption of maintenance therapy in the general population, potentially shorter duration of maintenance therapy, and our use of a conservative exclusion of SPMs diagnosed within 12 months of multiple myeloma diagnosis. The current study endeavored to estimate the contribution of high dose chemotherapy to SPM development, and thus addresses a different question than recent RCTs, and, lacking additional treatment data, we cannot assess the contribution of post-aHSCT lenalidomide maintenance to SPM development.

The current study has several limitations. All observational studies of treatment modalities are subject to selection bias. However, it is unlikely that physicians accounted for risk of SPM when offering aHSCT to patients, as relative contribution of aHSCT to the risk of SPM development is poorly defined. Second, the combined CCR-OSHPD data base does not include detailed treatment information, so we cannot account for various induction and maintenance strategies. The chemotherapy variable is applicable only to first course therapy, not later lines of therapy, and does not have additional details available. As lenalidomide maintenance after aHSCT, and the combination of low dose melphalan and lenalidomide, have been associated with SPM development, there is a potential for unmeasured confounding if patients undergoing aHSCT were more likely to receive lenalidomide maintenance^22,28,29,32^. However, practice patterns have indicated a rapid adoption of novel agents in the treatment of multiple myeloma, arguing that most patients would have had at least some exposure during the modern treatment eras to immunomodulatory agents^44,45^. Common use of post-aHSCT lenalidomide maintenance starting in 2012 may have resulted in increased SPM rates in the most recent time frame. The current data does not include detailed treatments, so this hypothesis cannot be directly tested, but deserves future analysis with more mature follow up and treatment data. While patients undergoing aHSCT may live longer, we addressed this potential source of survival time bias by accounting for the competing risk of death, treating aHSCT as a time dependent covariate in regression analyses, and excluding patients who died within a year of diagnosis. We also undertook a conservative approach to exclude SPMs diagnosed within 1 year of MM diagnosis to ensure that we did not count concurrent primaries, or asymptomatic malignancies identified during MM work-up or during initial treatment periods when oncologic follow-up is likely to be frequent. The CCR relies on death certificate data to assign a cause of death. This may be a source of attribution bias when considering cause-specific mortality. The current study also has several strengths. By including all multiple myeloma cases diagnosed in California, we have been able to compare rates of SPM development among myeloma patients undergoing a specific treatment with relatively long follow-up times, rather than comparing to the general population. To our knowledge using multiple myeloma patients as comparators, rather than the background population, to assess the association between aHSCT and SPM development is unique in the literature, and likely provides an estimation of the risk associated with aHSCT in these patients. California is one of the most populous and diverse states in the United States, likely increasing generalizability.

In conclusion, our study agrees with prior studies which have identified an increased risk of hematologic SPMs in multiple myeloma patients. The increased risk among aHSCT recipients compared to non-aHSCT recipients is an important consideration for patients undergoing the procedure. However, while the increased risk of hematologic SPMs is high in relative terms, the absolute risk increase over the subsequent 10 years is small, and, in our opinion, outweighed by the potential benefits to both overall and progression-free survival conferred by aHSCT use^8–12^. As MM patients continue to live longer, robust data on SPM risks associated with various treatment strategies are necessary for high quality, informed shared decision making.

## Supplementary information

Supplemental Table
