# Supplementary material for: Effect of autologous hematopoietic stem cell transplant on the development of second primary malignancies in multiple myeloma patients
Source: Blood Cancer J. 2021 Jan 7;11(1):5. doi: 10.1038/s41408-020-00400-4 (PMC7791054; doi:10.1038/s41408-020-00400-4)
Supplement: Supplementary file 1 — Supplemental Table [file 41408_2020_400_MOESM1_ESM.docx]

| **Supplemental Table S1: Baseline Demographics of the Matched* Cohorts** | | | | | | | |
| --- | --- | --- | --- | --- | --- | --- | --- |
|  | **All** |  | **aHSCT** |  | **No aHSCT** |  | **P-Value** |
| **Variables** | **N** | **%** | **N** | **col %** | **N** | **col %** |  |
| **All** | 6,020 | 100.0% | 2430 | 100.0% | 3,590 | 100.0% | . |
| **Gender** |  |  |  |  |  |  |  |
| Male | 3,596 | 59.7% | 1458 | 60.0% | 2,138 | 59.6% | 0.7294 |
| Female | 2,424 | 40.3% | 972 | 40.0% | 1,452 | 40.4% | 0.7294 |
| **Race/Ethnicity** |  |  |  |  |  |  |  |
| NH White | 4,010 | 66.6% | 1609 | 66.2% | 2,401 | 66.9% | 0.5907 |
| African-American | 564 | 9.4% | 234 | 9.6% | 330 | 9.2% | 0.5677 |
| Hispanic | 1,060 | 17.6% | 428 | 17.6% | 632 | 17.6% | 0.9931 |
| Asian/Pacific Islander | 374 | 6.2% | 153 | 6.3% | 221 | 6.2% | 0.8249 |
| Other/Unknown | 12 | 0.2% | 6 | 0.2% | 6 | 0.2% | 0.4959 |
| **Age at Diagnosis**** | 59 | 28 – 77 | 58 | 28 – 76 | 59 | 30 – 77 | <0.0001 |
| **Treatment Era of Diagnosis** |  |  |  |  |  |  |  |
| 1991-1997 | 725 | 12.0% | 288 | 11.9% | 437 | 12.2% | 0.7075 |
| 1998-2002 | 1,205 | 20.0% | 453 | 18.6% | 752 | 20.9% | 0.0283 |
| 2003-2007 | 1,682 | 27.9% | 713 | 29.3% | 969 | 27.0% | 0.0462 |
| 2008-2013 | 2,408 | 40.0% | 976 | 40.2% | 1,432 | 39.9% | 0.8302 |
| **1st Coarse of Treatment** |  |  |  |  |  |  |  |
| **Chemotherapy** |  |  |  |  |  |  |  |
| Yes | 4812 | 79.9% | 2271 | 93.5% | 2541 | 70.8% | <.0001 |
| No | 1,138 | 18.9% | 152 | 6.3% | 986 | 27.5% | <.0001 |
| Unknown | 70 | 1.2% | 7 | 0.3% | 63 | 1.8% | <.0001 |
| **Radiation** |  |  |  |  |  |  |  |
| Yes | 1,625 | 27.0% | 707 | 29.1% | 918 | 25.6% | 0.0025 |
| No | 4,394 | 73.0% | 1723 | 70.9% | 2,671 | 74.4% | 0.0027 |
| Unknown | 1 | 0.0% | . | . | 1 | 0.0% | 0.4106 |
| **nSES Status** |  |  |  |  |  |  |  |
| nSES 1-Lowest | 655 | 10.9% | 259 | 10.7% | 396 | 11.0% | 0.6491 |
| nSES 2 | 916 | 15.2% | 362 | 14.9% | 554 | 15.4% | 0.571 |
| nSES 3 | 1190 | 19.8% | 473 | 19.5% | 717 | 20.0% | 0.6279 |
| nSES 4 | 1,489 | 24.7% | 617 | 25.4% | 872 | 24.3% | 0.3312 |
| nSES 5-Highest | 1,770 | 29.4% | 719 | 29.6% | 1,051 | 29.3% | 0.7939 |
| **Insurance Coverage** |  |  |  |  |  |  |  |
| No insurance/Self Pay | 91 | 1.5% | 18 | 0.7% | 73 | 2.0% | <.0001 |
| Private Insurance | 3,659 | 60.8% | 1621 | 66.7% | 2,038 | 56.8% | <.0001 |
| Medicaid/Government | 536 | 8.9% | 200 | 8.2% | 336 | 9.4% | 0.1313 |
| Medicare | 1412 | 23.5% | 499 | 20.5% | 913 | 25.4% | <.0001 |
| Unknown Insurance | 322 | 5.3% | 92 | 3.8% | 230 | 6.4% | <.0001 |
| **Comorbidities† (within 2 years prior)** |  |  |  |  |  |  |  |
| Unknown/No admissions | 3,353 | 55.7% | 1346 | 55.4% | 2,007 | 55.9% | 0.6935 |
| 0 Comorbidities | 506 | 8.4% | 218 | 9.0% | 288 | 8.0% | 0.193 |
| 1-2 Comorbidities | 1217 | 20.2% | 502 | 20.7% | 715 | 19.9% | 0.4819 |
| ≥3 Comorbidities | 944 | 15.7% | 364 | 15.0% | 580 | 16.2% | 0.218 |
| aHSCT: autlogous stem cell transplant, nSES: neighborhood socioeconomic status  *Matching Procedure:  Each aHSCT recipient was matched with 1-2 non-aHSCT patients on sex, age +/- 3 years, year of diagnosis +/- 2 years, race/ethnicity, nSES quintile, Elixhauser comorbidity score (NA, 0, 1-2 or 3+)  **Age expressed as median and range  †Elixhauser Comorbidity Index ascertained from prior admissions. Patients therefore were NA if they had no prior admissions, 0, 1-2 or 3+. | | | | | | | |
